# Fusion Versus Non-fusion for Thoracolumbar Burst Fractures Treated With Short-Segment Posterior Instrumentation Including the Fracture Level

**DOI:** 10.7759/cureus.82331

**Published:** 2025-04-15

**Authors:** Sharif Jonayed, Abdullah Al Mamun Choudhury, Md. Ziaul Hasan, Md. Shah Alam

**Affiliations:** 1 Orthopaedics and Traumatology, National Institute of Traumatology and Orthopaedic Rehabilitation, Dhaka, BGD; 2 Spine Surgery, National Institute of Traumatology and Orthopaedic Rehabilitation, Dhaka, BGD; 3 Orthopaedic and Spine Surgery, National Institute of Traumatology and Orthopaedic Rehabilitation, Dhaka, BGD; 4 Spine Surgery, Bangladesh Spine and Orthopaedic Hospital, Dhaka, BGD

**Keywords:** fusion, incomplete neuro deficit, non-fusion, short segment posterior instrumentation, thoracolumbar burst fractures

## Abstract

Introduction

Thoracolumbar burst fractures are the most typical variant of injury to the spine. However, debate over the optimum treatment approach continues. Although spinal fusion has long been considered the gold standard in spinal fixation, the non-fusion procedure has recently gained prominence.

Objectives

To evaluate fusion versus non-fusion procedures in surgically treated thoracolumbar burst fractures.

Methods

This quasi-experimental study included 64 patients with single-level thoracolumbar fractures treated between July 2014 and June 2019. Patients were randomly allocated into two cohorts of 32 (50%) each based on registration numbers: those with odd numbers underwent fusion surgery (Group I), and those with even numbers underwent non-fusion surgery (Group II). All patients received short-segment posterior instrumentation, including the fractured vertebra. Radiological, functional, and neurological outcomes were assessed and contrasted. SPSS version 25.0 (IBM Corp., Armonk, NY) was used to conduct the statistical analysis.

Results

No statistically significant difference was observed between the two groups in terms of radiological outcomes, functional outcomes, neurological improvement, or implant failure rates. The non-fusion group, however, exhibited reduced operative duration and hemorrhage, absence of donor site morbidity, and preserved a greater number of motion segments. No notable intraoperative or postoperative complications were noted, nor were any substantial differences observed in either group.

Conclusion

Routine spinal fusion may be unnecessary for surgically repaired thoracolumbar burst fractures, as the non-fusion method provides similar outcomes with additional advantages.

## Introduction

Thoracolumbar burst fractures are the most common form of spinal injury, accounting for over 50% of thoracolumbar trauma and comprising 10-20% of all vertebral injuries [1]. The disorder primarily affects younger people and is frequently associated with neurological impairment and kyphotic deformity, which greatly impairs their physical capabilities. However, the best course of treatment is still up for debate on issues like conservative versus operative treatments, anterior versus posterior approaches, short versus long segment fixation, and fusion versus non-fusion techniques [2-4]. Conservative treatment is frequently advised for patients with normal neurology, which may result in the loss of correction for their kyphotic angle or spinal height [5]. On the other hand, there is a possibility that the symptoms at the most recent follow-up would not correlate with the increased residual deformity [6]. There may be certain procedure-related problems associated with the actual surgical treatment [7]. Therefore, it is very important to take into account all the factors, including the patient's characteristics, the morphology of the fracture, and their neurological condition. Operative treatment is the most effective approach for managing any fracture that is unstable and accompanied by neurological dysfunction. The objective is to alleviate pressure on the neural tissue, ensuring stability and realigning the spine, while also promoting early mobilization for optimal patient rehabilitation [8].

The goal can be readily accomplished through meticulous patient selection for posterior instrumentation with short-segment pedicle screws and rods [9-12]. Regardless of implant failure, numerous studies have demonstrated the importance of fusion in improving fracture-fixing longevity and reducing the chance of losing kyphosis correction [13-20]. In contrast, some studies demonstrated positive results without fusion [21, 22]. Hence, we performed a prospective quasi-experimental study to examine the results of treating thoracolumbar burst fractures using short-segment pedicle fixation, with or without fusion. The hypothesis of the present study proposed that fusion is not essential for the treatment of certain patients with thoracolumbar burst fractures undergoing short-segment pedicle screw fixation.

## Materials and methods

The study was performed at Bangladesh Spine and Orthopedic Hospital, a tertiary-level hospital in Dhaka, Bangladesh, from July 2014 to June 2019 with the Institutional Ethical Board Committee (Ref. BSOH/Academic/Research/2014/0012). 

A total of 64 patients with a single-level AO type A3 thoracolumbar burst fracture (T11 to L2) and an incomplete neurological deficit, who had a load-sharing score of less than 6 and a kyphotic angle greater than 20° or a reduction in vertebral body height greater than 50% within three weeks of the injury, were included in the study. The study excluded patients who had a pathological fracture, pedicle fracture in the index vertebra, polytrauma, were either younger than 16 years or older than 65 years, had a history of previous spine surgery, or had a simultaneous head injury.

**Figure 1:**
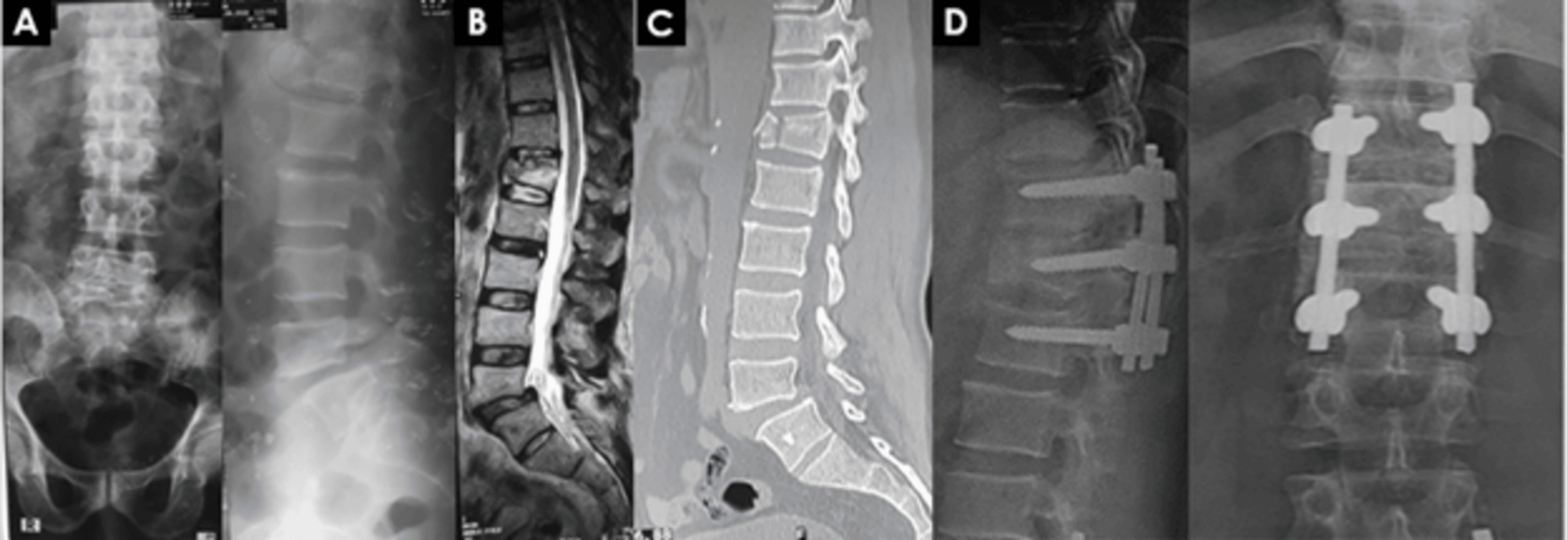
61-year-old male with burst #L1 with ASIA-C neurology, treated with short-segment posterior fixation including fractured vertebra and posterolateral fusion (Patient-1). X-ray reveals that a 61-year-old male (burst #L1 with ASIA-C neurology) who underwent short-segment posterior fixation, which included vertebral fracture and posterolateral fusion. Panels A–D report the following: A: Pre-operative X-ray, lumbo-sacral spine (AP and lateral view) B: MRI of LS spine (T2 sagittal section) C: CT scan of LS spine (sagittal section) D: Final follow-up at three years showing good fusion Image Credit: Sharif Jonayed

**Figure 2:**
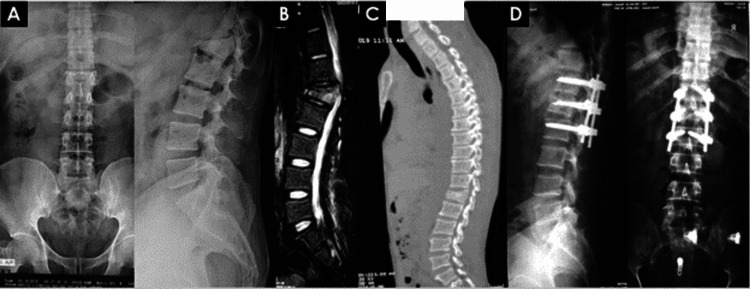
42-year-old male with burst #L1 with ASIA-C neurology, treated with short-segment posterior fixation including fractured vertebra without fusion (Patient 2). The treatment for a 42-year-old male with burst #L1 and ASIA-C neurology involved short-segment posterior fixation, which included the fractured vertebra (Figure 2) without fusion. Panels A–D demonstrate the following: A: Pre-operative X-ray, lumbo-sacral spine (AP and lateral view) B: MRI of LS spine (T2 sagittal section) C: CT scan of LS spine (sagittal section) D: Final follow-up at three years Image Credit: Sharif Jonayed

A comprehensive written agreement was obtained from all the patients, explicitly outlining the specifics of the surgery and the various therapy methods involved. Following the initial resuscitation, according to the ATLS protocol, a comprehensive history was obtained, including information about the individual's gender, age, cause of injury, any existing medical conditions, and occupation. Additionally, a thorough examination was conducted. The patient's neurological condition was evaluated using the American Spinal Injury Association (ASIA) impairment scale [23]. Subsequently, the patients were segregated into two cohorts using a random allocation procedure, where Group I was assigned to patients with odd numbers, and Group II was assigned to patients with even numbers. Those in Group I underwent posterior fixation and fusion (Figure 1), while those in Group II received fixation alone (Figure 2).

A typical posterior midline technique was used in all patients, along with a short-segment transpedicular fixation. This involved applying fixation screws one level above and below the injured segment, as well as one or two additional screws to promote lordosis. Fluoroscopic guidance was used throughout the entire procedure, including in the frontal and lateral planes. When necessary, the pedicle screw construct was moved to correct the spinal deformity. If an injury is detected in the pedicle of the index vertebra, then the corresponding side is excluded for the insertion of the screw. Decompression was performed when needed. The screws were fastened using two rods that were shaped into a lordotic form. Before tightening the set screws, a distraction force was exerted under the injured endplate.

**Figure 3:**
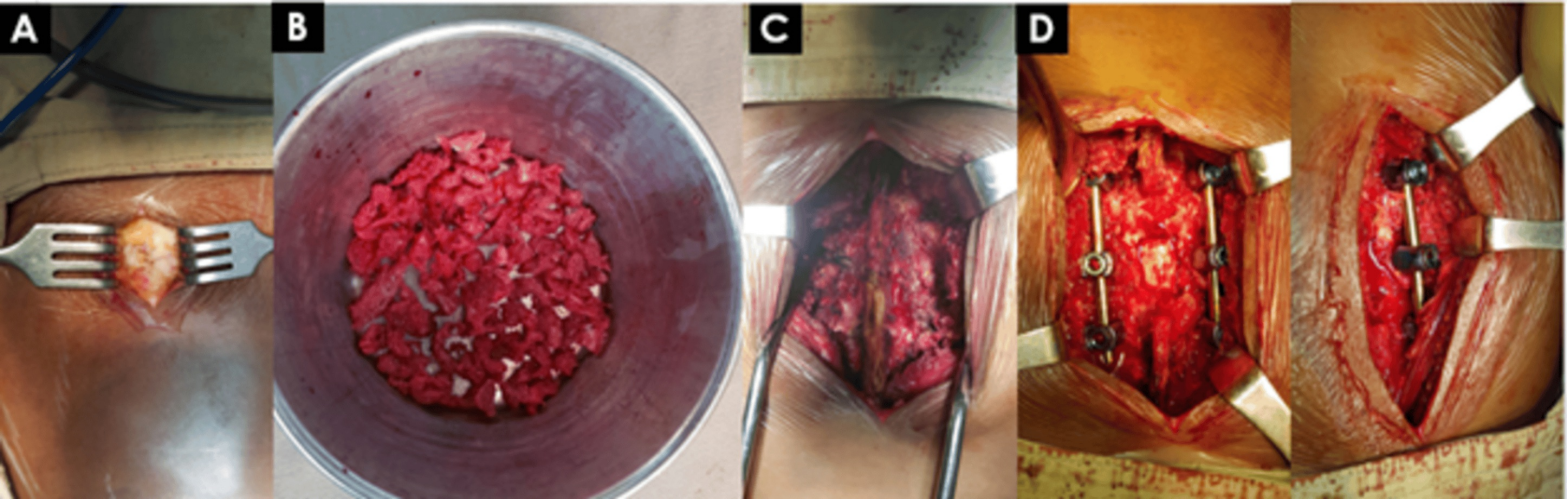
Postero-lateral fusion. The posterior fusion procedure was completed after the outer layers of bone from the posterior structures were removed. An autologous bone graft was harvested from the posterior iliac crest and packed back into the bleeding bone. Panels A–D demonstrate the following: A: Exposure of posterior iliac crest B: Bone chips taken from posterior iliac crest C-D: Postero-lateral inter-transverse fusion Image Credit: Sharif Jonayed

The posterior fusion procedure was performed following the removal of the outer layer of bone from the posterior structures. The autologous bone graft was obtained from the posterior iliac crest and placed on the bleeding bone (Figure 3).

The non-fusion group did not utilize any graft material. Ultimately, the incision was sealed using the customary technique, although a suction drainage tube was left in place. The intraoperative data, encompassing surgical duration, blood loss, and intraoperative adverse events, were meticulously documented. Following the surgery, patients were strongly encouraged to engage in early mobilization while wearing a thoracolumbar brace for six weeks. Engaging in excessive physically demanding tasks and leaning forward were strictly forbidden. All perioperative medical or surgical problems were documented, which refers to any occurrence requiring a specific intervention or therapy.

Patients underwent evaluations at regular intervals of one, three, six, and twelve months post-surgery, followed by annual assessments. During each visit, an impartial observer who had no affiliation with the treatment evaluated radiological parameters and functional outcomes. The local kyphosis angle was determined on lateral plain radiographs using the Cobb method [24]. The determination of kyphosis correction loss involved calculating the difference between the local kyphosis angle recorded at the latest follow-up and the local kyphosis angle measured following the initial correction procedure. The patients' fusion status was primarily assessed using plain radiographs, following the classification system of Christensen et al. [25]. The definition of solid fusion encompasses the existence of uninterrupted and well-developed osseous trabeculae connecting the intersegmental fusion zone while restricting flexion-extension movement to under 5 mm. In cases where a distinct fusion was not observed, fine-cut computed tomography scans accompanied by reconstructions were incorporated. The neurological status was assessed using the ASIA impairment scale, whereas using the Greenough Low-Back Outcome Score, the functional outcomes were noted [26] during the most recent follow-up.

The statistical analysis was conducted using the SPSS for Windows statistical software, version 25.0 (IBM Corp., Armonk, NY). Variable parameters were compared at various follow-up times between the fusion and non-fusion groups using the independent sample t-test for continuous data and the chi-square test for categorical data. To find out if the injured vertebral body height and kyphotic angle will change over time, the repeated-measure analysis of variance (ANOVA) test was utilized. A statistically significant difference was found at a significance level of p < 0.05.

## Results

The fundamental demographic characteristics are shown in Table 1. There was no statistically significant difference between the two groups. The patient's age (p = 0.239), gender distribution (p = 0.391), and load-sharing score (p = 0.086) were comparable in both groups. In both groups, the most prevalent level of damage was L1, followed by D12. In terms of neurological status, the group with the highest number of cases was represented by ASIA D, followed by ASIA C in both categories. The average duration from the occurrence of the injury to the surgical procedure was 10.41 ± 2.92 in Group I and 10.34 ± 2.93 in Group II (p = 0.932). The average duration of follow-up was 32.78 ± 5.43 months in group I and 33.66 ± 3.82 months in Group II.

**Table 1:** Demographic characteristics between two groups The values are presented as mean ± standard deviation (SD) or counts (n). Statistical comparisons between the two groups are denoted by p-values.

Variable		Group I, fusion (n = 32)	Group II, non-fusion (n = 32)	P-value
Age in years (mean ± SD)		37.25 ± 10.61	40.91 ± 13.80	0.239
Sex (male/female)		30/2 (93.75/6.25)	28/4 (87.5/12.5)	0.391
Mechanism of injury, n(%)	Fall from height	23 (71.88)	17 (53.125)	0.121
	Road traffic injury	9 (28.12)	15 (46.875)	
Skeletal level of injury n(%)	D11	2 (6.25)	4 (12.50)	0.835
	D12	10 (31.25)	9 (28.12)	
	L1	16 (50.00)	16 (50.00)	
	L2	4 (12.50)	3 (9.38)	
Load-sharing score (mean ± SD)		4.47 ± 1.34	3.37 ± 0.941	0.086
ASIA impairment scale, n(%)	B	4 (12.50)	3 (9.38)	0.111
	C	6 (18.75)	8 (25.00)	
	D	22 (68.75)	21 (65.62)	

The radiographic parameters are displayed in Table 2. Both groups demonstrated improvement in terms of the average kyphotic angle and average anterior vertebral height. An independent sample t-test showed no statistically significant differences between the two groups at any time interval, except for the pre-operative decreased anterior VBH, which was statistically significant.

**Table 2:** Radiological parameters between the two groups The data are shown as counts (n) or mean ± standard deviation (SD). Parameters that cannot be measured or are not applicable are denoted by hyphens (-). The anterior vertebral body height (VBH) is measured in millimeters (mm), while kyphotic angles are measured in degrees (°). *Pre-operative decreased anterior VBH is statistically significant (p=0.017).

Variable	Group I, fusion (n = 32)	Group II, non-fusion (n = 32)	P-value
Mean preoperative kyphotic angle (^0^) (mean ± SD)	21.53 ± 3.69	21.81 ± 5.08	0.806
Mean postoperative kyphotic angle (^0^) (mean ± SD)	2.97 ± 2.09	3.25 ± 2.72	0.644
Follow up kyphotic angle (^0^) (mean ± SD)	13.03 ± 2.39	13.00 ± 2.95	0.963
Lost kyphotic angle (^0^) (mean ± SD)	9.88 ± 2.08	9.69 ± 1.57	0.641
preoperative decreased anterior VBH in mm (mean ± SD)	46.56 ± 10.95	39.94 ± 10.67	0.017*
Postoperative decreased anterior VBH in mm (mean ± SD)	16.50 ± 4.24	14.97 ± 4.04	0.143
Follow-up decreased anterior VBH in mm (mean ± SD)	24.34 ± 5.59	21.78 ± 5.53	0.070
Screw breakage, n(%)	7 (21.87)	12 (37.5)	0.171
Solid fusion, n(%)	27 (84.37)	-	-

Table 3 indicated that there were no statistically significant differences among the groups in evaluating neurological recovery as measured by the ASIA Scale (chi-square p = 0.171). The Low-Back Outcome Score in the fusion group was 49.41 ± 11.57, while in the non-fusion group it was 54.13 ± 10.53 (p = 0.093). Group I observed successful fusion in 27 (84.4%) individuals. Pain at the site of the donation was observed in all instances within the fusion group.

**Table 3:** Comparison of neurological and functional status between two groups The data are shown as counts (n) or mean ± standard deviation (SD). A hyphen ("-") denotes that the data is either unavailable or not applicable.

Scale	Group I, fusion (n = 32)		Group II, non-fusion (n = 32)		P-value
	Preoperative	Postoperative	Preoperative	Postoperative	
ASIA Scale, n(%)					0.171
B	4 (12.5)	-	3 (9.375)	-	
C	6 (18.75)	-	8 (25)	-	
D	22 (68.75)	-	21 (65.625)	12 (37.5)	
E	0	25	0	20	
Low-Back Outcome Scale at final follow-up (mean ± SD)	-	49.41 ± 11.57	-	54.13 ± 10.53	0.093

However, the non-fusion group demonstrated significantly shorter operative time and lower blood loss compared to the fusion group (p = 0.000). The average duration of the surgical procedure was 223.75 ± 19.13 minutes for the fusion group and 151.88 ± 17.90 minutes for the non-fusion group (Table 4). The mean estimated blood loss was 519.38 ± 58.86 ml and 347.81 ± 30.85 ml for patients in the fusion and non-fusion groups, respectively (Table 4).

**Table 4:** Operative variables The data are presented as the mean ± standard deviation (SD) or as counts (n). Parameters that are either not applicable or not reported are denoted by hyphens (-).

Variable	Group I, fusion (n = 32)	Group II, non-fusion (n = 32)	P-value
Mean duration from injury to surgery (day) (range)	10.41 ± 2.92	10.34 ± 2.93	0.932
Mean blood loss (ml) (mean ± SD)	519.38 ± 58.86	347.81 ± 30.85	<0.001
Mean operative time (min) (mean ± SD)	223.75 ± 19.13	151.88 ± 17.90	<0.001
Total hospital stays (days) (mean ± SD)	10.66 ± 2.35	9.84 ± 1.55	-
Mean follow-up (months) (mean ± SD)	32.78 ± 5.43	33.66 ± 3.82	-
Donor site pain, n(%)	32 (100)	-	-

Both groups had screw breakage; however, a crosstab analysis revealed no statistically significant difference. No significant problems were observed in any of the patients, save for cerebrospinal fluid leaks in two patients from the fusion group and one instance of implant failure in the non-fusion group caused by a fall.

## Discussion

While the selection of treatment for thoracolumbar burst fractures has not yet been standardized, surgery is consistently beneficial for unstable spines with neurological deficits. The inclusion criteria in our study procedure are well established. Our goal was to attain a spine with proper realignment to provide mechanical stability using a short-segment pedicle screw fixation, which includes one or two intermediate screws at the affected vertebra. Additionally, we aimed to determine if simultaneous fusion is required or not.

Pedicle screws offer robust three-column stability, exhibiting exceptional resistance to pull-out and cut-out forces, enabling them to endure substantial stress levels. However, they are incapable of averting anterior collapse, particularly in a severely fragmented fracture. The occurrence of implant failure accompanied by recurrent kyphosis in the latter stages is a significant concern. Anterior column reconstruction is mandatory for load-sharing scores greater than 6. We deliberately omitted these cases. Nevertheless, our study revealed a notable progression of regional kyphosis in both the non-fusion and fusion groups over time. The average loss of correction in the fusion group was 9.88 ± 2.08, while it was 9.69 ± 1.57 in the non-fusion group. The observed change did not reach statistical significance (p = 0.641). This is further corroborated by multiple studies [1, 22, 27]. Defino and Canto [28] observed a continued increase in kyphosis during the final follow-up, even with the use of posterolateral fusion and transpedicular bone grafts to stop kyphosis from worsening in the future. These results suggest that increasing kyphosis may be inevitable even after fusion and that the residual deformity did not match the symptoms observed over the follow-up period [9].

Both the fusion and non-fusion groups showed a significant improvement in anterior vertebral body height with measurements of 7.84 ± 2.48 and 7.25 ± 2.58, respectively. Nevertheless, the outcome did not demonstrate statistical significance (p = 0.594) and was similar to the findings of Tezeren et al. [29]. Wang et al. [22] demonstrated a notable disparity in the preservation of correction of anterior vertebral body height (VBH) in the non-fusion group. The fusion group exhibited a notably larger percentage of vertebral body height loss, potentially due to partial damage to the posterior column (including supraspinous, interspinous ligaments, and spinous processes) during the posterior fusion procedure. This may have resulted in heightened instability of the spinal column, transitioning from a two-column to a three-column injury, thereby leading to a more significant decrease in vertebral body height during the subsequent evaluation. However, in the non-fusion group, the decrease in correction can mostly be attributable to the collapse of the damaged disc, as also shown by Sanderson et al. [21].

Because of our investigation, we feel that the progressive loss of disc height at the injured disc plays a more essential role in worsening kyphosis than the mild degree of decreased vertebral body height. According to Wang et al. [22], the primary factor leading to the loss of correction is the compression of the disc space.

The fusion group exhibited significantly increased mean intraoperative blood loss and mean operative time, as supported by previous studies [1, 22, 27, 29].

The Low Back Outcome Score did not show a statistically significant difference between the groups (p = 0.093), which is consistent with the findings of prior studies by Wang et al. [22] and Jindal et al. [27]. Both groups exhibited similar neurological outcomes when compared to previous investigations. However, no additional therapy was necessary as the patients did not exhibit any noticeable clinical symptoms. We observed the occurrence of seven instances of screw breakage in the non-fusion group, while in the fusion group there were five instances. Screw breakage is a rather common occurrence in the treatment of burst fractures, typically found within the first six months before the fracture has fully healed. This is the time when there is a higher likelihood of recurrent kyphosis. Patients in the fusion group experienced discomfort at the donor site. The benefits of non-fusion were the eradication of difficulties associated with the donor site [21].

In both groups, we did not regularly extract the screws. Elective removal of the implants might be performed for cultural reasons, as part of a therapy procedure, or due to implant failure. Hardware can still malfunction even after fusion has been accomplished. Implant failure may occur despite successful fusion due to persistent flexural loading [20]. Bone mineral density (BMD) was not regularly conducted to determine the severity of osteoporosis in both groups, which could have significantly impacted the stability and degree of corrective loss.

Study limitation

The study population is not large enough to draw a conclusion on a broader population; as a result, the sample may not be sufficiently randomized or diverse.

## Conclusions

In our study, we found that fusion offers no added benefit above the non-fusion group in surgically treated thoracolumbar burst fractures. Furthermore, the fusion patients had longer surgical times, more blood loss, and greater discomfort at the donor site. So, fusion may not be appropriate as a routine standard procedure for the stabilization of thoracolumbar fractures. Non-fusion techniques provide adequate treatment with less operative time, fewer complications, and less patient discomfort, thus emphasizing that pertinent decisions and the choice between techniques are important components of clinical outcomes and patient satisfaction. Non-fusion strategies may be preferable in certain cases to enhance therapeutic efficacy and patient safety, particularly when less invasive approaches are appropriate.
